# Explaining infant feeding: The role of previous personal and vicarious experience on attitudes, subjective norms, self‐efficacy, and breastfeeding outcomes

**DOI:** 10.1111/bjhp.12254

**Published:** 2017-06-23

**Authors:** Naomi C. Bartle, Kate Harvey

**Affiliations:** ^1^ Centre for Technology Enabled Health Research Coventry University UK; ^2^ School of Psychology and Clinical Language Sciences University of Reading UK

**Keywords:** breastfeeding, infant feeding, past experience, SCT, self‐efficacy, TPB, vicarious experience

## Abstract

**Objectives:**

Breastfeeding confers important health benefits to both infants and their mothers, but rates are low in the United Kingdom and other developed countries despite widespread promotion. This study examined the relationships between personal and vicarious experience of infant feeding, self‐efficacy, the theory of planned behaviour variables of attitudes and subjective norm, and the likelihood of breastfeeding at 6–8 weeks post‐natally.

**Design:**

A prospective questionnaire study of both first‐time mothers (*n* = 77) and experienced breastfeeders (*n* = 72) recruited at an antenatal clinic in South East England.

**Methods:**

Participants completed a questionnaire at 32 weeks pregnant assessing personal and vicarious experience of infant feeding (breastfeeding, formula‐feeding, and maternal grandmother’s experience of breastfeeding), perceived control, self‐efficacy, intentions, attitudes (to breastfeeding and formula‐feeding), and subjective norm. Infant feeding behaviour was recorded at 6–8 weeks post‐natally. Multiple linear regression modelled the influence of vicarious experience on attitudes, subjective norm, and self‐efficacy (but not perceived control) and modelled the influence of attitude, subjective norm, self‐efficacy, and past experience on intentions to breastfeed. Logistic regression modelled the likelihood of breastfeeding at 6–8 weeks.

**Results:**

Previous experience (particularly personal experience of breastfeeding) explained a significant amount of variance in attitudes, subjective norm, and self‐efficacy. Intentions to breastfeed were predicted by subjective norm and attitude to formula‐feeding and, in experienced mothers, self‐efficacy. Breastfeeding at 6 weeks was predicted by intentions and vicarious experience of formula‐feeding.

**Conclusion:**

Vicarious experience, particularly of formula‐feeding, has been shown to influence the behaviour of first‐time and experienced mothers both directly and indirectly via attitudes and subjective norm. Interventions that reduce exposure to formula‐feeding (perhaps by limiting advertising) or cushion mothers from its effects may enable more mothers to meet their breastfeeding goals.

**Statement of contribution**

***What is already known on this subject?***

- Rates of breastfeeding in the United Kingdom are low and resistant to change.
- Self‐efficacy may be an important and modifiable factor for breastfeeding initiation and maintenance.

***What does this study add?***

- Self‐efficacy may only be a relevant factor among mothers who already have personal experience of breastfeeding.
- Vicarious experience of formula‐feeding has been shown to be related to a lower rate of breastfeeding at 6 weeks.

## Background

The health and economic benefits of breastfeeding for both child and mother are well documented and have been recently highlighted in an important Breastfeeding Series in the Lancet (Rollins *et al*., 2016; Victora *et al*., 2016). However, the United Kingdom has some of the lowest breastfeeding rates in the developed world with recent figures showing the initiation of breastfeeding at 81%, followed by a sharp decline after birth. Less than 1% of mothers were still breastfeeding exclusively at 6 months as recommended by the WHO (McAndrew *et al*., 2012; Word Health Organisation, 2002).

Qualitative research has identified diverse social and attitudinal factors that influence the infant feeding decision (Andrew & Harvey, 2010; Hoddinott & Pill, 1999; McFadden & Toole, 2006; Stewart‐Knox, Gardiner, & Wright, 2003). Mothers commonly report embarrassment and concern about breastfeeding, and restricted feelings of independence preventing them from incorporating breastfeeding into their lifestyle. Notably, these issues are important for both first‐time and multiparous mothers, as even those with experience of breastfeeding feel inhibited about going out with their older children (Andrew & Harvey, 2010; Stewart‐Knox *et al*., 2003). Many mothers report stopping breastfeeding earlier than intended, citing practical or physical difficulties such as pain, difficulty with the infant's latch, and concerns about milk supply – problems that could be prevented with appropriate education and support (McAndrew *et al*., 2012). Earlier‐than‐intended cessation is often associated with feelings of guilt and may contribute to the development of post‐natally depression (Borra, Iacovou, & Sevilla, 2014; Lee, 2007; Marshall, Godfrey, & Renfrew, 2007), indicating the importance of understanding the psychosocial factors involved in breastfeeding to provide appropriate support and reduce the related inequalities in health.

Much of the research that has attempted to quantify the psychosocial and cognitive factors associated with infant feeding has been based on either the theory of planned behaviour (TPB; Ajzen, 1991, 2002; Ajzen & Fishbein, 2005) and its predecessor, the theory of reasoned action (TRA; Fishbein, 1979), or Bandura's social cognitive theory (SCT). The TPB posits that behaviour is directly determined by an intention to perform the activity and that without intention a behaviour is unlikely to occur. Intention is formed through a combination of attitudes, subjective norm (perceived approval from important others), and perceived behavioural control (PBC) with PBC able to also influence behaviour directly (Ajzen, 1991; Ajzen & Fishbein, 2005). Within the model, attitude and subjective norms can only influence behaviour through their contribution to intention formation. Attitude, subjective norm, and PBC (‘TPB constructs’) are each the product of specific outcome beliefs and evaluations of the importance of those outcomes, as formed by past experiences and influenced by socio‐demographic characteristics (Ajzen, 1991). Recently, the TPB has been heavily criticized as (i) a theory which is difficult to falsify, (ii) one that is not valid; that is, it does not provide a good explanation of behaviour and many of the assertions of the original model (e.g., that relationships between attitudes and behaviour are completely mediated by intentions) have not been supported; and (iii) one that is no longer useful (Odgen, 2003; Sniehotta, Presseau, & Araujo‐Soares, 2014). Applications of the TPB and the TRA to infant feeding have reported significant cross‐sectional associations between attitudes, norms, perceived control, and breastfeeding intentions and suggest that intentions are strongly related to actual breastfeeding rates (most recently, Cabieses, Waiblinger, Santorelli, & McEachan, 2014; Lawton, Ashley, Dawson, Waiblinger, & Conner, 2012; McMillan *et al*., 2009, 2008). Most studies measured intentions after giving birth, so the measure may be conflated by breastfeeding experience. However, one which measured intentions before giving birth found that they were a strong predictor of behaviour, with *r* = .67 for breastfeeding initiation and *r* = .42 for breastfeeding at 6 weeks (McMillan *et al*., 2008). Other prospective studies have shown direct associations (not mediated by intentions) between breastfeeding behaviour and attitudes, subjective norms, and PBC/self‐efficacy (Duckett *et al*., 1998; Lawton *et al*., 2012; Manstead, Plevin, & Smart, 1984; Manstead, Proffitt, & Smart, 1983; McMillan *et al*., 2008). Therefore, there is evidence that the components of the TPB are relevant to understanding infant feeding intentions and behaviour, although they may not be best operationalized in the TPB model.

A further criticism of the TPB is that it does not offer strategies for changing behaviour, other than by addressing the beliefs that are hypothesized to underlie attitudes, subjective norms, and perceived control. In contrast, Bandura's SCT posits that self‐efficacy is important for motivation to perform a behaviour and persistence with that behaviour in the face of difficulties *and* it identifies four methods for increasing self‐efficacy (personal mastery, vicarious experience, verbal persuasion, and emotional arousal) (Bandura, 1977, 1986). Breastfeeding‐specific self‐efficacy, assessed either prenatally or up to 1 week after giving birth, predicts a higher likelihood of breastfeeding up to 6 months post‐natally and may be an important and modifiable variable to consider in terms of breastfeeding promotion (Bailey, Clark, & Shepherd, 2008; Blyth *et al*., 2002; Dennis, 2003; Dennis & Faux, 1999). SCT explains that self‐efficacy is gained primarily through personal experience of overcoming difficulties (‘mastery’). Consistent with this, there is evidence that breastfeeding self‐efficacy is higher among mothers with previous, positive, breastfeeding experiences (McCarter‐Spaulding & Dennis, 2010; Otsuka *et al*., 2013). In the absence of personal mastery (as in the case of first‐time mothers), Bandura suggests that vicarious experience – seeing others successfully mastering the skill – can also promote self‐efficacy (Bandura, 1977, 1986). As breastfeeding cannot be practised until the baby is born, yet has to be mastered quickly to satisfy the needs of the infant, vicarious experience may be an important route for promoting successful maintenance of breastfeeding. Interviews with young, low‐income mothers revealed that vicarious experience (seeing other women breastfeeding) is important as a means of acquiring ‘embodied knowledge’ (Hoddinott & Pill, 1999). The authors suggested that embodied knowledge of the act of breastfeeding instils confidence in new mothers and helps establish breastfeeding. Conversely then, an absence of vicarious experience may contribute to the practical difficulties with breastfeeding that mothers often report, such as difficulty with the infants’ latch, painful breastfeeding, and concerns about milk supply (Andrew & Harvey, 2010; McAndrew *et al*., 2012). This also opens up the possibility of encouraging contact between pregnant and breastfeeding women, to increase vicarious experience of breastfeeding and promote self‐efficacy in order to help mothers sustain breastfeeding (Hoddinott *et al*., 2009).

Self‐efficacy has been a well‐utilized theory for examining the factors associated with breastfeeding, and there have been positive outcomes from trials of interventions based on increasing it (McQueen, Dennis, Stremler, & Norman, 2011; Nichols, Schutte, Brown, Dennis, & Price, 2009; Otsuka *et al*., 2013). However, these trials vary in the degree to which they describe the components of the intervention and how those components relate to Bandura's four suggested pathways for increasing self‐efficacy. There has been much less attention paid to how each specific component may be best utilized to increase self‐efficacy. There has been very little research examining the influence of past experience upon breastfeeding self‐efficacy itself, or examining the relationship between breastfeeding self‐efficacy and breastfeeding outcomes independently of past experience (i.e., in a sample of first‐time mothers). Furthermore, there has been little exploration of the role of vicarious experience as a potential modifier of self‐efficacy and breastfeeding outcomes.

This study aimed to prospectively examine the relationships between previous infant feeding experience (both direct breastfeeding experience and vicarious breastfeeding and formula‐feeding experience), breastfeeding self‐efficacy, attitudes to breastfeeding and formula‐feeding, subjective norm, breastfeeding intentions, and actual breastfeeding at 6 weeks post‐partum. Specifically, the study aimed to answer the following research questions:

1. Do vicarious experience of infant feeding and direct personal experiences of breastfeeding influence breastfeeding self‐efficacy and/or attitudes, subjective norms, and PBC?
2. Does infant feeding experience influence intentions to breastfeed, over and above the attitudes, subjective norms, PBC, and self‐efficacy?
3. Does infant feeding experience influence actual breastfeeding at 6 weeks, over and above the intentions, attitudes, subjective norms, PBC, and self‐efficacy?
4. Does breastfeeding self‐efficacy contribute to the prediction of breastfeeding intentions and behaviour over and above PBC, attitudes, and subjective norms, and does this differ between mothers with and without previous breastfeeding experience?

The data for these analyses are drawn from a larger, prospective longitudinal study investigating multiple factors associated with infant feeding for which pregnant women completed questionnaires on four occasions (12–15 weeks and 32 weeks pregnant; 6 weeks and 6 months after giving birth). The data reported here were collected on the second (32 weeks pregnant) and third (6 weeks post‐natally) occasions. Complete data are recorded elsewhere (Andrew, 2010).

## Method

The project proposal was approved by the local research ethics committee (NHS; Ref. 07/Q1602/60) and the institutional research ethics committee.

### Participants

**Figure 1:**
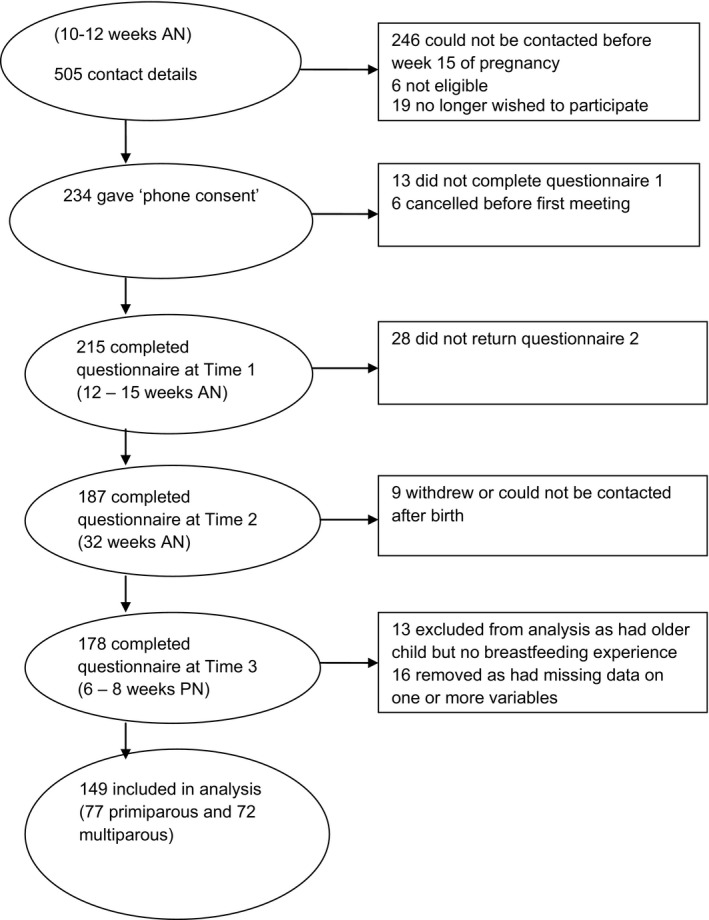
Flow diagram showing sample attrition. AN = antenatal; PN = post‐natally.

Sonographers approached pregnant women attending for their dating scan (approximately 10–12 weeks of pregnancy) at a UK hospital. After reading a brief description of the study, women interested in participating were invited to provide contact details. The researcher (NB) telephoned potential participants, explained the study in full, and offered to visit them. Women were eligible if they had a positive scan outcome, they were able to understand spoken and written English, and had no physical or mental disability that would prevent them from completing the questionnaire. Both primi‐ and multiparous women were eligible. This study reports data from 149 women who provided complete data both when 32 weeks pregnant and 6 weeks after the birth. Figure 1 shows the recruitment and attrition rates.

### Procedure

After initial recruitment by telephone, participants were visited by the researcher (NB) in their homes or another convenient location to obtain written consent and obtain initial data (not reported here, except ‘previous difficulty breastfeeding’). Approximately 31 weeks into their pregnancy, participants were contacted again by telephone and another questionnaire was sent to them to complete and return by post. The researcher identified when participants had given birth using hospital records. Approximately 5 weeks after birth, the researcher contacted participants and arranged to visit them to complete the third questionnaire. Table 1 summarizes the data collection. At all times, participants were able to respond by post if they preferred. When posting questionnaires, the researcher (NB) made up to two reminder telephone calls. When communicating with participants, and within the questionnaires themselves, we aimed to balance the representation of breast‐ and formula‐feeding, and to avoid being perceived as endorsing either method of feeding.

**Table 1:** Timetable of data collection

Time point	Measures
T1: 12–15 weeks pregnant (visit)	Demographics (age, ethnicity, education) Parity Difficulty breastfeeding (multiparous mothers only)
T2: 32 weeks pregnant (post)	Attitude to breastfeeding Attitude to formula‐feeding Subjective norm Perceived behavioural control Self‐efficacy Vicarious experience of breastfeeding Vicarious experience of formula‐feeding Grandmother's experience of breastfeeding Breastfeeding intentions
T3: Approx. 6–8 weeks post‐natally (visit)	Breastfeeding initiation Any breastfeeding at 6 weeks

### Measures

#### Demographics

Participants recorded their date of birth, ethnicity, and highest level of education, and the age and feeding method of any older children.

#### Psychosocial variables

Participants responded to all questionnaire items except vicarious experience using 100‐mm visual analogue scales (VAS) with labels at each end. VAS were employed because they provide continuous data, and allow participants greater flexibility. There is evidence that, compared to Likert scales, VAS data are more variable, more uniformly distributed, and more sensitive to change (Brunier & Graydon, 1996). The vicarious experience items had 3‐ or 5‐point response scales representing the level of exposure (e.g., never, sometimes, often). These scores were converted to numerical scores (1–5) for analysis with higher scores indicating more exposure (see Table 2 for full details).

**Table 2:** Questionnaire measures and scale reliability

Measure	Source	Scales (means were calculated from the following items, except for Subjective Norm and Intention which used different formula as detailed below)	Cronbach's α	Min; Max
Attitude to breastfeeding	Manstead *et al*. (1983)	Unpleasant–pleasant; embarrassing–not embarrassing; unhealthy–healthy; unattractive–attractive; inconvenient–convenient; unnatural–natural	.76	0;100
Attitude to formula‐feeding	Manstead *et al*. (1983)	As above. Participants answered the same scales separately for breast and formula‐feeding	.76	0;100
Subjective norm	Similar to Manstead *et al*. (1983)	Four referents: ‘people involved in my maternity care’, ‘the baby's father’, ‘my own mother’, and ‘my friends with children’ Participants stated the degree to which each referent thought they should breastfeed or formula‐feed, and how important that referent's opinion was to their decision‐making. For each referent a score was computed which was their endorsement of breastfeeding minus their endorsement of formula‐feeding, multiplied by the importance score (which had been divided by ten to reduce the range of the scale)	.67	−1,000;+1,000
Perceived behavioural control	Ajzen (2002)	How much control do you feel you have over your decision about feeding your baby? (no control–complete control)’ How I feed my baby is completely up to me (strongly disagree–strongly agree)	.83	0;100
Breastfeeding self‐efficacy	Dennis (2003)	Breastfeeding Self‐Efficacy Scale Short Form with three items removed to enable completion by women with low intentions to breastfeed. Eleven items, that is, ‘I will always determine that my baby is getting enough milk’ (not at all confident–always confident)	.95	0;100
Vicarious experience of formula‐feeding	n/a	How often have you seen [someone/relative or close friend] giving their baby formula milk? How often have you fed someone else's baby with formula milk? 5‐point scale: ‘never (1)’ to ‘very often (5)’; scores from three items summed	.81	3;15
Vicarious experience of breastfeeding	n/a	How frequently have you seen [someone/relative or close friends] breastfeeding their baby? 5‐point scale: ‘never’ to ‘very often’; scores from two items summed	.81	2;10
Grandmother's experience of breastfeeding	n/a	How much did your mother breastfeed her children? (Not at all (1), Sometimes (2), or Always (3))	n/a	1;3
Previous difficulty breastfeeding	n/a	Mothers who had breastfed before rated their previous experience using six items about how painful, time‐consuming, and difficult breastfeeding was, as well as how easy it was for the infant to latch onto the breast, how confident the mother was in her supply of milk, and how much these experiences influenced her intentions for feeding her new baby	.85	0:100

Participants were given a questionnaire which comprised measures of self‐efficacy, vicarious experience of infant feeding (vicarious experience of breastfeeding, vicarious experience of formula‐feeding, and grandmother's experience of breastfeeding; see Table 2), and personal breastfeeding experience and items consistent with the TPB (namely attitudes to breastfeeding, attitudes to formula‐feeding, subjective norms, and perceived behavioural control). Individual TPB items were created following the guidelines set out by Conner and Sparks (2005) and Ajzen (2006) and were informed by qualitative research (e.g., Andrew & Harvey, 2010; Hoddinott & Pill, 1999) and previous applications of the TPB to breastfeeding (Manstead *et al*., 1983, 1984; Swanson & Power, 2005). Questions were asked in relation to either breastfeeding or formula‐feeding with definitions of each at the beginning of the questionnaire. Participants were asked to respond to the questions in relation to feeding the baby they were currently expecting. No duration of breastfeeding or other context information was specified except for the intention items which were specified in terms of 4 weeks and 6 months. Principal components analysis (PCA) was used to construct composite variables and eliminate uninformative items. Internal reliability was checked with Cronbach's alpha. Mean scores were calculated for each scale with the exceptions of vicarious experience, subjective norm, and intention (for calculations, see Table 2 and below for Intention). Full details of each measure, scale items and reliability, and scale calculations are provided in Table 2.

#### Breastfeeding outcomes

Intention: Participants rated the strength of their intention to exclusively breastfeed, and formula‐feed for 4 weeks and for 6 months after birth (total four items). ‘Breastfeeding Intentions’ were calculated as the sum of the two exclusive breastfeeding items, minus the sum of the two exclusive formula‐feeding items which resulted in the range −200 to +200. At the post‐natally visit, participants were asked whether they had ever breastfed their new baby, and if so, whether they were still breastfeeding. Breastfeeding at 6 weeks was dichotomized into ‘still breastfeeding’ and ‘no longer breastfeeding’. Participants were considered to be breastfeeding if they were giving any breast milk at this age, regardless of supplementation with formula milk, in line with the Public Health Outcomes Framework indicator of prevalence of breastfeeding at 6–8 weeks after birth.

### Statistical analysis

Analyses were carried out separately for primiparous and multiparous women. Women who had older children but had never breastfed them were excluded, as they represented a small group (*n* = 13) that could confound the influence of quality of breastfeeding experience in which we were particularly interested. Linear regression was employed to investigate the influence of all vicarious experience variables (and difficulty breastfeeding in the multiparous sample) upon the TPB variables (attitudes, subjective norm, and self‐efficacy). Regression models were then run to predict the outcome variables (prenatal intentions, and breastfeeding at 6 weeks) from TPB and experience variables. To restrict the number of predictors, univariate correlations were inspected and potential predictors were retained only if their absolute correlation with the dependent variable was .3 or greater. Hierarchical linear regression analyses were carried out to predict breastfeeding intentions first from TPB variables alone, and then with experience variables added. Hierarchical logistic regression was employed to predict breastfeeding at 6 weeks, from intentions alone and from intentions plus other TPB and experience variables.

## Results

Descriptive statistics and univariate correlations between potential predictor and outcome variables are shown in Table 3. The mean age of the first‐time mothers was 31 years and of experienced mothers was 33 years. Most participants (71%) had been educated to degree level, and 87% described their ethnicity as White. As ethnicity was not associated with breastfeeding status in our data, we did not include ethnicity in further analyses. Almost all of the sample (99%) initiated breastfeeding, and the majority of the sample were still breastfeeding their infants at 6 weeks – slightly more among experienced breastfeeders (86%) than among first‐time mothers (73%) (see Table 4). Mean scale scores fell above the mid‐point for attitude to breastfeeding, subjective norm in favour of breastfeeding, and intentions to breastfeed. Scores reflected the full range of vicarious experience and past difficulty breastfeeding. Mean scores were around the mid‐point on attitude to formula‐feeding and breastfeeding self‐efficacy, with self‐efficacy being slightly higher among experienced breastfeeders than among first‐time mothers.

**Table 3:** Descriptives, and univariate correlations with intentions to breastfeed and breastfeeding at 6 weeks

Correlated with:	Min; Max	Primiparous *n* = 77			Multiparous *n* = 72		
		*r*		Mean (*SD*)	*r*		Mean (*SD*)
		Intention to breastfeed	Breastfeeding at 6 weeks		Intention to breastfeed	Breastfeeding at 6 weeks	
Age (years)	19; 47	.03	.15	31.22 (5.31)	.08	−.04	33.21 (4.93)
Education	1; 4	.10	.04	1.74 (0.44)	.20	.23	1.67 (0.47)
Vic. exp. breastfeeding	3; 10	.14	−.02	6.29 (2.01)	.27*	.25*	6.92 (1.96)
Vic. exp. of formula	3; 15	−.09	−**.35** **	10.14 (3.16)	−.28*	−**.33** **	10.81 (2.47)
Grandmother's experience	1; 3	**.40** **	.08	2.30 (0.84)	.06	**.33** **	2.35 (0.79)
Attitude to breastfeeding	38; 100	**.32** **	−.06	74.26 (11.97)	.29*	.18	76.45 (13.62)
Attitude to formula‐feeding	2.2; 99.8	−**.45** **	−**.37** **	55.79 (12.79)	−**.55** **	−**.43** **	54.44 (18.27)
Subjective norm	−147.1; 746.1	**.45** **	−.20	236.06 (171.20)	**.47** **	**.31** **	195.20 (176.11)
PBC	4.5; 100	.20	−.08	75.71 (21.91)	.04	−.26*	88.00 (12.42)
Self‐efficacy	4.9; 95.9	.18	.09	49.89 (16.53)	**.50** **	**.36** **	60.46 (21.98)
Difficulty breastfeeding	0.2; 90.5	n/a	n/a	n/a	−**.39** **	−**.39** **	39.54 (22.35)
Intention to breastfeed	−162.00; 200.00	–	**.33** **	126.90 (62.35)	–	**.45** **	135.50 (73.73)

Notes

Text in bold indicates criterion for inclusion as a predictor for outcomes has been met (absolute correlation ≥ .3).

*p < .05, **p < .01.

**Table 4:** Infant feeding outcomes by first‐time and experienced breastfeeders

Sample	*N*	Initiated breastfeeding	At 6 weeks post‐natally		
			Exclusive breastfeeding	Mixed feeding	Exclusive formula‐feeding
First‐time mothers	77	76 (98.7%)	26 (33.8%)	30 (39.0%)	21 (27.3%)
Experienced breastfeeders	72	72 (100%)	31 (43.1%)	31 (43.1%)	10 (14.0%)

Inspection of correlations between demographics (age and education) and all other variables showed no strong relationships involving the demographics (*r*s −.21 to .28; details not shown), so age and education were excluded from all regression models.

PBC over breastfeeding was correlated with breastfeeding self‐efficacy, strongly in the primiparous sample (*r *=* *.48) and moderately in the sample of experienced breastfeeders (*r *=* *.27). PBC over breastfeeding showed no correlation with intentions or breastfeeding behaviour for the primiparous sample. In the mothers with previous breastfeeding experience, PBC showed no correlation with Intention, but a negative correlation with behaviour (indicating those with more perceived control were less likely to breastfeed). This correlation was significant, but too small to meet our criteria for inclusion. In contrast, self‐efficacy was strongly correlated with intention and behaviour in the multiparous sample (Table 3). As the correlations indicated that self‐efficacy was a better predictor than PBC, and to avoid multicollinearity, self‐efficacy was used rather than PBC in all relevant models.

### Experience as a predictor of self‐efficacy, attitudes, and subjective norm (Question 1)

Among first‐time mothers (Table 5), the three vicarious experience variables together accounted for 12%, 18%, and 11% of the variance in attitude to breastfeeding, attitude to formula‐feeding, and subjective norm in favour of breastfeeding, respectively, but did not explain a significant proportion of the variance in breastfeeding self‐efficacy. The most prominent individual predictor was their own mothers’ breastfeeding experience (maternal grandmother's experience) which significantly influenced all four variables (attitudes to breastfeeding and formula‐feeding, subjective norm in favour of breastfeeding, and breastfeeding self‐efficacy). More vicarious experience of formula‐feeding predicted a more positive attitude to formula‐feeding, but not any other variables. Vicarious experience of breastfeeding did not predict any of the variables.

**Table 5:** First‐time mothers’ (*n* = 77) prediction of TPB variables from three Experience variables

	TPB dependent variable			
	Attitude to breastfeeding^a^	Attitude to formula‐feeding^b^	Subjective norm^a^	Self‐efficacy^a^
Predictor	Beta [95% CI]	Beta [95% CI]	Beta [95% CI]	Beta [95% CI]
Vicarious experience of breastfeeding^c^	.10 [−0.12; 0.32]	−.04 [−0.25; 0.17]	.18 [−0.04; 0.40]	.12 [−0.11; 0.35]
Vicarious experience of formula‐feeding^d^	−.08 [−0.14; 0.30]	.37 [0.16; 0.58]**	−.15 [−0.37; 0.08]	.01 [−0.25; 0.21]
Maternal grandmother's experience of breastfeeding^c^	.37 [0.15; 0.58]***	−.31 [−0.51; −0.10]**	.33 [0.12; 0.55]**	.29 [0.07; 0.51]*
Model adjusted *R* ^2^	.12**	.18***	.11**	.06

Notes

a Higher score = more favourable towards breastfeeding.

b Higher score = more favourable towards formula‐feeding.

c Higher score = more experience of breastfeeding.

d Higher score = more experience of formula‐feeding.

*p < .05, **p < .01, ***p < .001 95% CI = 95% confidence interval.

Among mothers with previous breastfeeding experience (Table 6), the four experience variables together explained a significant and substantial proportion of the variance in attitude to breastfeeding (29%) and breastfeeding self‐efficacy (47%), and a significant proportion of the variance in subjective norm in favour of breastfeeding (11%) but did not explain attitude to formula‐feeding. Previous difficulty breastfeeding predicted attitude to breastfeeding and breastfeeding self‐efficacy but not subjective norm in favour of breastfeeding or attitude to formula‐feeding. More vicarious experience of breastfeeding predicted a more positive attitude to breastfeeding, and less positive attitude to formula‐feeding. Maternal grandmother's experience of breastfeeding predicted subjective norm. Vicarious experience of formula‐feeding did not predict any of the variables.

**Table 6:** Experienced mothers’ (*n* = 72) prediction of TPB variables from four Experience variables

	TPB dependent variable			
	Attitude to breastfeeding^a^	Attitude to formula‐feeding^b^	Subjective norm^a^	Self‐efficacy^a^
Predictor	Beta [95% CI]	Beta [95% CI]	Beta [95% CI]	Beta [95% CI]
Vicarious experience of breastfeeding^c^	.20 [0.00; 0.41]*	−.29 [−0.52; −0.05]*	.19 [−0.04; 0.41]	−.07 [−0.25; 0.10]
Vicarious experience of formula‐feeding^d^	.03 [−0.17; 0.23]	.09 [−0.14; 0.33]	−.03 [−0.26; 0.19]	−.04 [−0.22; 0.13]
Maternal grandmother's experience of breastfeeding^c^	−.06 [−0.27; 0.15]	.05 [−0.19; −0.29]	.31 [0.08; 0.55]*	−.09 [−0.27; 0.09]
Difficulty breastfeeding^e^	−.53 [−0.74; −0.32]***	.04 [−0.21; 0.29]	−.02 [−0.26; 0.22]	−.71 [−0.89; −0.52]***
Model adjusted *R* ^2^	.29***	.04	.11*	.47***

Notes

a Higher score = more favourable towards breastfeeding.

b Higher score = more favourable towards formula‐feeding.

c Higher score = more experience of breastfeeding.

d Higher score = more experience of formula‐feeding.

e Higher score = more difficult experience.

*p < .05, **p < .01, ***p < .001.

### Predicting breastfeeding intentions (Question 2)

In first‐time mothers, three TPB variables (attitude to breastfeeding and formula‐feeding, and subjective norm) and maternal grandmother's experience of breastfeeding met the criterion for inclusion in the model for Intentions (correlation |*r*| ≥ .3; see Table 3). Thirty‐four per cent of variance in first‐time mothers’ breastfeeding intentions was explained by this model which is shown in Table 7. A positive attitude to formula‐feeding predicted less intention to breastfeed, while subjective norm in favour of breastfeeding predicted more intention to breastfeed.

**Table 7:** Linear regression models predicting Intentions to Breastfeed

Predictor	Sample	
	First‐time mothers (*n* = 77) Beta [95% CI]	Experienced mothers (*n* = 72) Beta [95% CI)
Attitude to breastfeeding^a^	.17 [−0.03; 0.37]	Not in model
Attitude to formula‐feeding^b^	−.29 [−0.50; −0.09]**	−.34 [−0.54; −0.14]**
Subjective norm^a^	.26 [0.05; 0.46]*	.25 [0.06; 0.45]*
Self‐efficacy^a^	Not in model	.36 [0.18; 0.54]*
Grandmother’s experience of breastfeeding^c^	.17 [−0.04; 0.38]	Not in model
Model *R* ^2^	.34***	.47***

Notes

a Higher score = more favourable towards breastfeeding.

b Higher score = more favourable towards formula‐feeding.

c Higher score = more experience of breastfeeding.

*p < .05, **p < .01, ***p < .001.

In mothers with previous breastfeeding experience, attitude to formula‐feeding, subjective norm in favour of breastfeeding, breastfeeding self‐efficacy, and difficulty breastfeeding met the criterion for inclusion in the model for predicting breastfeeding intentions. However, breastfeeding self‐efficacy and difficulty breastfeeding were too highly correlated for them both to remain (tolerance values for both = .46), so the regression was rerun excluding difficulty breastfeeding (as past experience is hypothesized to influence intentions via self‐efficacy). The final model (Table 7) explained 47% of variation in intentions. Attitude to formula‐feeding was a negative predictor, while subjective norm in favour of breastfeeding and breastfeeding self‐efficacy were positive predictors of breastfeeding intentions.

### Predicting breastfeeding behaviour at 6 weeks post‐partum (Questions 3 and 4)

Intentions alone explained 15% and 26% of variance in first‐time and experienced mothers’ breastfeeding rates at 6 weeks, respectively (Table 8). Among first‐time mothers, attitude to formula‐feeding and vicarious experience of formula‐feeding were strongly correlated with behaviour, meeting the criterion for inclusion in the model. For mothers with breastfeeding experience, many variables (six in all; Table 3) met the criterion for inclusion. In both samples, when these additional variables were added, the variance explained increased significantly to 32% (first‐time mothers) and 68% (experienced mothers). However, in both samples breastfeeding intention became non‐significant. As the aim of the analysis was to determine what predicted behaviour over and above the known predictive effect of intentions, the inclusion in the model of many variables that were correlated with breastfeeding intention undermined that aim. Therefore, the analysis was rerun with only intentions plus the variables that were correlated with breastfeeding but not intentions.

**Table 8:** Logistic regression models predicting Breastfeeding at 6 weeks

Predictor	Sample	
	First‐time mothers (*n* = 78) OR [95% CI]	Experienced mothers (*n* = 72) OR [95% CI)
Intention to breastfeed	1.01 [1.00;1.02]**	1.01 [1.01;1.02]**
Model 1 Nagelkerke *R* ^2^	0.15**	0.26**
Intention to breastfeed	1.01 [1.00;1.02]**	1.01 [1.00;1.02]*
Vicarious experience of formula‐feeding^a^	0.73 [0.60; 0.91]**	0.63 [0.40; 0.99]*
Grandmother’s experience of breastfeeding^b^	Not in model	4.50 [1.36;14.86]*
Model 2 Nagelkerke *R* ^2^	0.30***	0.50**

Notes

OR = odds ratio.

a Higher score = more experience of formula‐feeding.

b Higher score = more experience of breastfeeding.

*p < .05, **p < .01, ***p < .001.

In first‐time mothers, the final model included intentions and vicarious experience of formula‐feeding. This model explained 30% of the variance in breastfeeding at 6 weeks, and both predictors were significant. In mothers with previous breastfeeding experience, the final model explained 50% of the variance in breastfeeding at 6 weeks and all predictors were significant (see Table 8).

**Figure 2:**
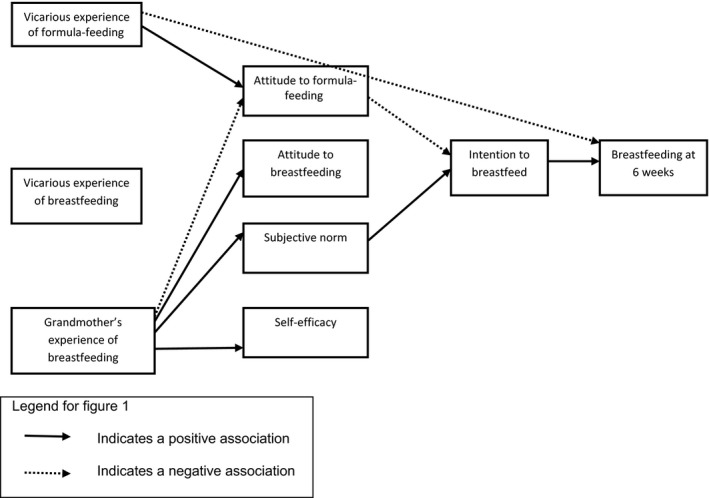
Modelling infant feeding decisions in first‐time mothers.

**Figure 3:**
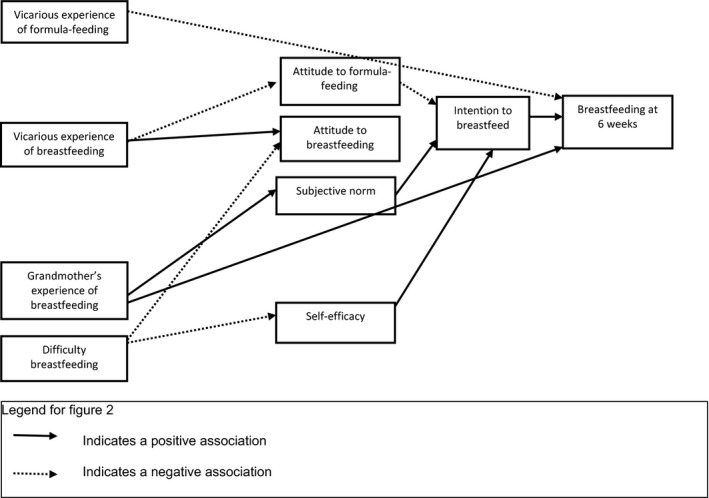
Modelling infant feeding decisions in experienced mothers.

Figures 2 and 3 summarize all the foregoing findings for first‐time mothers and mothers with previous breastfeeding experience, respectively.

## Discussion

### Summary of findings

This study aimed to explore the relationships between previous experience (personal direct experience of breastfeeding and vicarious experience of infant feeding), breastfeeding self‐efficacy, attitudes, subjective norm, and perceived control and breastfeeding at 6–8 weeks. We tested whether past experience could account for some of the variance in breastfeeding self‐efficacy, infant feeding attitudes, and subjective norms. Then, we explored whether self‐efficacy and past experience of breastfeeding contributed to the prediction of breastfeeding intentions and breastfeeding at 6–8 weeks.

A novel finding is that among both first‐time and experienced mothers, vicarious experience of infant feeding predicted attitudes and subjective norms that were also in favour of breastfeeding, although not breastfeeding self‐efficacy. Pro‐breastfeeding descriptive norms have been shown to be important in the prediction of breastfeeding at 10 days (McMillan *et al*., 2009), but this study showed that vicarious experience may act in the formation of social cognitions such as attitudes. Vicarious experience of formula‐feeding was associated directly with behaviour; those mothers with more vicarious experience of formula‐feeding were less likely to be breastfeeding at 6–8 weeks regardless of their own personal experience. For experienced mothers, their own past difficulty breastfeeding predicted a less favourable attitude to breastfeeding and lower self‐efficacy confirming previous findings that the nature of previous experience influences self‐efficacy (Otsuka *et al*., 2013). This study is also the first to demonstrate that although high breastfeeding self‐efficacy was associated with stronger intentions to breastfeed, this was only the case among mothers with their own previous breastfeeding experience.

Our findings confirm the importance of attitude (in this case attitude to formula‐feeding) and subjective norm for infant feeding, both of which predicted intentions in first‐time mothers and experienced breastfeeders. However, it is notable that despite strong positive attitudes to breastfeeding, these were not associated with intentions or behaviour; it was attitudes to formula‐feeding that had a stronger influence over behaviour. We found no relationship between perceived control and either intentions or behaviour and did not include this variable in the regression models. This may reflect a weakness of our particular measure, so that questions were interpreted as experiencing control over aspects of feeding (i.e., timing/location) rather than reflecting control over the decision/ability to breastfeed, or the self‐efficacy questions were simply a better reflection of participants’ beliefs about managing those situations. We also found a strong relationship between intentions and breastfeeding status at 6 weeks, although we are the first to identify that this relationship is much stronger for women who have breastfed before than for first‐time mothers.

### The role of self‐efficacy in infant feeding intentions and behaviour

Among experienced mothers, self‐efficacy was a predictor of intentions. This supports Bandura's SCT that increased breastfeeding self‐efficacy is associated with a higher intention/motivation to breastfeed. However, in this analysis we have not shown that self‐efficacy contributes to the maintenance of breastfeeding at 6–8 weeks over and above intentions, which SCT would predict. In our data, self‐efficacy, intentions, and attitudes were all very highly correlated, which suggests that our measures were accessing one construct (which perhaps reflects on past behaviour) rather than three separate constructs as intended.

There was a striking contrast between first‐time mothers and experienced breastfeeders in the role of self‐efficacy, which was completely uncorrelated with either intentions or behaviour in first‐time mothers. As far as the authors can identify, this is the first time the BSES‐SF, measured antenatally, has been used to predict intentions or behaviour among mothers with no prior breastfeeding experience. Dennis’ work to date has focussed on the predictive utility of the BSES‐SF after giving birth. An exception is Blyth *et al*. (2002) which tested the predictive utility of the BSES‐SF measured in the last trimester weeks on breastfeeding outcomes at 1 week and 4 months post‐natally, but this was a mixed sample (including first‐time and experienced mothers). It is possible that apparent failure of the BSES‐SF to predict breastfeeding intentions of behaviour that is reported here is due to the changes made to the scale (three items from the original 14 were removed). However, as the BSES‐SF did show strong predictive utility over both intentions and behaviour in the experienced sample, it seems more likely to represent a difference between first‐time and experienced mothers. Perhaps first‐time mothers do not have enough information to form realistic expectations of breastfeeding in order to make accurate predictions of their ability to cope with the demands. Schwarzer (2014) argues that self‐efficacy can only be optimistic self‐beliefs based on personal experience, not unrealistic expectations. Likewise, Ajzen (1991) suggests that PBC needs to be based on enough information to be realistic before it can influence behaviour. Alternatively, perhaps first‐time mothers are less influenced by their perceived ability to breastfeed than they are by social and cultural influences or their self‐efficacy over formula‐feeding which has not been accounted for in this study. Future research might investigate this further, perhaps by exploring perceptions of self‐efficacy in the late antenatal period, and/or development of a tool to examine formula‐feeding self‐efficacy in order to draw comparisons with breastfeeding self‐efficacy in the prediction of infant feeding behaviours.

### The role of vicarious experience in infant feeding attitudes, self‐efficacy, intentions, and behaviour

Attitudes, subjective norms, and self‐efficacy were all predicted by at least one of the vicarious experience variables, indicating that vicarious experience may be one of the pathways to the formation of these social cognitions. It is interesting that vicarious experience of breastfeeding was not associated with any other variable among first‐time mothers, and for those with prior breastfeeding experience, it was associated with attitudes but not with self‐efficacy. This therefore does not provide any support for attempting to increase breastfeeding self‐efficacy, and therefore breastfeeding, by increasing vicarious experience – for example, with exposure to more breastfeeding role models. It is important to note that this was the first time our measure of vicarious experience had been employed, and it may be that it did not cover the full range or extent of vicarious experience in enough detail to show a predictive effect. Future research might wish to develop a more robust measure of vicarious experience of breastfeeding and/or attempt to distinguish between vicarious experience and descriptive norms before the role of vicarious experience of breastfeeding can be dismissed. Vicarious experience of formula‐feeding appeared to have a direct influence over behaviour – over and above intentions. This variable is likely to represent social or cultural norms surrounding infant feeding. High levels of vicarious experience of formula‐feeding indicate a formula‐feeding culture in which breastfeeding is not given much consideration (therefore directly influencing behaviour). As well as accessing vicarious experience, this variable may also tap into a descriptive social norm (reflecting the predominant behaviour in a social group). This is in contrast to the subjective norm questions which assess the perceived attitudes (rather than perceived behaviour) of others. Descriptive norms have been indicated as a potential additional predictor in the TPB (Ajzen, 2006), and this finding provides further evidence for the distinction. This reflection of social norms would go some way to explaining the influence of this variable on attitudes and norms, but not necessarily why it has a direct influence on behaviour. To understand this, it is important to consider infant feeding not simply as a choice to breastfeed or not, but also whether to give formula‐feeds or not (or indeed a choice to combine the two). It is possible that in a society where formula‐feeding is both common practice and frowned upon, seeing other women giving formula‐feeds may offer support for this choice in cases where mothers are experiencing breastfeeding difficulties. Evidence suggests that once formula milk is introduced, duration of breastfeeding is shortened (possibly due to a reduction in milk supply; Howel & Ball, 2013; McAndrew *et al*., 2012). Therefore, vicarious experience of formula‐feeding may ‘allow’ mothers to offer formula‐feeds, which in turn reduces their likelihood of continuing to breastfeed at 6–8 weeks. Although we did not specifically ask about exposure to formula milk advertising, this finding adds support to calls to control/limit formula milk advertising in order to protect mothers from the apparently powerful effect that exposure to formula‐feeding has on breastfeeding outcomes.

The maternal grandmother's experience of breastfeeding was associated with subjective norm, attitudes, and self‐efficacy in first‐time mothers, and with subjective norm and behaviour in experienced mothers. Like vicarious experience of formula‐feeding, this variable is also likely to reflect something more than vicarious experience; attitudes may pass down through generations and daughters may identify with their mothers and expect to have similar (positive or negative) experiences. Of course, it is not possible to modify the experience of maternal grandmothers today, but interventions could encourage parents to consider why their own mothers might not have breastfed and to explore how to manage conversations about breastfeeding with them in order to foster their support.

### Strengths of the study

This study is the first to directly investigate the role of previous personal and vicarious experience among the determinants of infant feeding method. Previous evidence suggested that the TPB variables (attitude, subjective norm, and PBC) and antenatal breastfeeding self‐efficacy would be related to breastfeeding outcomes (intentions and behaviour), but it was not known that these effects would be so much stronger for mothers with previous experience of breastfeeding than for first‐time mothers. Breastfeeding is an interesting test case for the role of past experience as it is a behaviour that cannot be practised before the need to feed a new baby (i.e., it has a definite start point). This means it is a behaviour of which some mothers have no personal experience, although they may have vicarious experience. This opportunity enabled us to establish that self‐efficacy in particular may need to be based on direct personal experience of a behaviour to predict future behaviour. When examining infant feeding, it is important to consider both breastfeeding and formula‐feeding as potentially competing behaviours. In contrast to previous studies, we have attempted to ask about many of the variables in relation to both behaviours, which has allowed us to demonstrate that attitudes to formula‐feeding and vicarious experience of formula‐feeding are important determinants of breastfeeding behaviour, with important implications for promoting and protecting breastfeeding. In future, we recommend building on this work to examine the role of self‐efficacy and/or perceived behavioural control for formula‐feeding as a potential avenue for deepening our understanding of how mothers make their decisions about infant feeding.

### Limitations

There are a number of limitations to the study. The sample mostly represents White, middle‐class mothers who are older and more likely to be breastfeeding than the wider population of mothers in the United Kingdom. However, even in this sample there was a considerable reduction in the rate of breastfeeding in the first 6 weeks (from 99% to 79%), and identifying the important factors associated with those who continue or stop breastfeeding by this stage is important. Furthermore, where the variables and analyses were similar to previous studies, the findings were also similar – even when compared to a deprived sample (McMillan *et al*., 2008, 2009). This suggests that many of the factors associated with infant feeding, and in particular the relative importance of past experience and self‐efficacy, may be similar across socio‐economic groups. The variables representing vicarious experience of infant feeding types are likely to represent the social norms in the culture in which the mother lives, and therefore, replicating this study with a more varied sample has the potential to show an increased influence of these variables. Having split the sample by parity for the analyses, this does leave small numbers in the regression analyses; however, where conclusions have been drawn regarding a lack of association, the effect sizes are so small that they are unlikely to be the result of low power. Finally, although drawing on a longitudinal design has provided stronger evidence of causality in terms of the predictors of breastfeeding at 6 weeks, many of the relationships tested were cross‐sectional, and the direction of causality or the absence of a confounding variable should not be assumed.

### Recommendations for policy, practice, and future research

Attitudes to formula‐feeding and vicarious experience of formula‐feeding showed direct relationships with intentions and/or behaviour, even when mothers had strong positive attitudes to breastfeeding. This suggests that pro‐breastfeeding messages are reaching mothers, but the prevailing behaviour of formula‐feeding may lead mothers to also hold positive beliefs about formula‐feeding. Interventions may need to tackle positive beliefs about formula‐feeding as well as any negative beliefs about breastfeeding in order to increase breastfeeding rates. This may require further prospective, qualitative research to identify which specific beliefs or cognitions about formula‐feeding are most influential and/or most receptive to change, and to identify any role of formula‐feeding self‐efficacy in the infant feeding decisions. Attitudes already formed by 32 weeks of pregnancy are influential over behaviour, so attempts to influence attitude should be made earlier; further prospective research may be necessary to determine the timing of this (e.g., earlier in pregnancy, or even pre‐pregnancy). Subjective norms in favour of breastfeeding were associated with stronger intentions to breastfeed, so a community‐ or population‐wide approach including fathers/partners, other family members, and health professionals in infant feeding discussions may be more effective than an individual approach to behaviour change. At a policy level, restrictions on the advertising of formula milk may be justified to protect mothers in the early weeks of breastfeeding. For mothers who have previous experience of breastfeeding, health professionals could encourage them to focus on their previous successes with breastfeeding, and perhaps reflect on any previous difficulty they encountered, supporting them to overcome it in the new pregnancy. The aim would be to maximize their self‐efficacy for breastfeeding a future child. Future research could explore the effectiveness of this approach.

### Conclusion

Vicarious experience of formula‐feeding, representing the social norms around infant feeding, has a direct negative influence over breastfeeding at 6 weeks in both first‐time and experienced mothers. Furthermore, attitudes to formula‐feeding (but not strong, positive attitudes to breastfeeding) and subjective norms were related to infant feeding intentions indicating that infant feeding intentions and behaviours may be more influenced by the prevalent behaviour in society (i.e., formula‐feeding) than by personal attitudes. Future research could explore this link further, with a view to understanding how we might start to break down the formula‐feeding culture in favour of breastfeeding.

Previous positive personal experience of breastfeeding enhances self‐efficacy, and this may relate to more positive intentions to breastfeed. However, for increased self‐efficacy to be translated into greater intentions and actual breastfeeding, it may need to be based upon realistic expectations of breastfeeding. Mothers with experience of breastfeeding may benefit from a focus on their previous successes to maximize their breastfeeding self‐efficacy for breastfeeding a subsequent child. With regard to recent criticisms of the TPB, these findings show that some of its predictor variables (attitude, intention) remain relevant, but the operation of the model does not fully capture the complex social and cultural determinants of competing infant feeding behaviours.

## Conflict of Interest

All authors declare no conflict of interest.
